# Field efficacy and safety of fluralaner solution for administration in drinking water for the treatment of poultry red mite (*Dermanyssus gallinae*) infestations in commercial flocks in Europe

**DOI:** 10.1186/s13071-017-2390-3

**Published:** 2017-10-09

**Authors:** Emmanuel Thomas, Mathieu Chiquet, Björn Sander, Eva Zschiesche, Annie Sigognault Flochlay

**Affiliations:** 1 MSD Animal Health Innovation GmbH, Zur Propstei 55270, Schwabenheim, Germany; 2 MSD Animal Health Innovation SAS, 7 rue Olivier de Serres, CS 67131, 49071 Angers Technopole, Beaucouzé France; 3 Merck Animal Health, 2 Giralda Farms, Madison, NJ USA

**Keywords:** Chicken, Clinical, *Dermanyssus gallinae*, Efficacy, Fluralaner, Poultry red mite

## Abstract

**Background:**

Welfare concerns, production losses caused by *Dermanyssus gallinae*, the poultry red mite (PRM), and widespread mite resistance to environmentally applied acaricides continue to drive an urgent need for new and effective control measures. Fluralaner is a novel systemic acaricide developed to address that need. A series of field studies was initiated to investigate the safety and efficacy of a fluralaner solution (10 mg/ml) administered in drinking water at a dose rate of 0.5 mg/kg on two occasions with a 7-day interval, for treatment of natural PRM infestations in chickens.

**Methods:**

Blinded, negative-controlled studies were completed in Europe across eight layer, two breeder, and two replacement chicken farms. At each farm, two similar flocks were housed in similar PRM-infested units (either rooms within a building, or separate buildings) varying from 550 to 100,000 birds per unit. One unit at each farm was allocated to fluralaner treatment, administered in drinking water on Days 0 and 7. One unit remained untreated. Mite traps were placed throughout each unit on Days -1, 0 or 1, 3, 6, 9, and 13 or 14, then at weekly or two-weekly intervals, retrieved after 24 h and processed for mite counts. Efficacy at each farm was assessed by mean PRM count reductions from traps in treated units compared with those from control units. Production parameters and safety were also monitored.

**Results:**

Efficacy was 95.3 to 99.8% on Day 3 and 97.8 to 100% on Day 9, thereafter remaining above 90% for 56 to 238 days after treatment initiation. Post-treatment improvement in egg-laying rate was greater by 0.9 to 12.6% in the treated group at 9 of the 10 layer or breeder farms. There were no treatment-related adverse events.

**Conclusion:**

Fluralaner administered at 0.5 mg/kg via drinking water twice, 7 days apart, was well tolerated and highly efficacious against the PRM in naturally infested chickens representing a range of production types and management systems. The results indicate that this novel treatment has potential to be the cornerstone of an integrated approach to reducing or eliminating the welfare and productivity costs of this increasingly threatening pest.

## Background

The poultry red mite (PRM), *Dermanyssus gallinae,* reduces bird welfare while causing substantial production losses (particularly in laying hens), acts as a vector of disease-causing pathogens, presents an occupational hazard for poultry-house workers and is increasingly a cause of medical concern for human populations living near poultry houses [1–10]. Recent surveys have confirmed the extremely high, increasing prevalence of PRM infestations in Europe, with an average of 83% of European layer houses infested, and up to 94% in The Netherlands, Germany and Belgium [4, 7]. Infestations with the PRM are found in all production types, from backyard or organic farms to intensive, enriched cage or barn systems [4]. The negative effects of PRM infestations on bird welfare and production are likely to be exacerbated by recent regulatory requirements which, by discontinuing use of conventional cages, may increase mite refugia while making such refugia more difficult to access with sprays and dusts. Additionally, the upcoming ban on beak trimming may also increase PRM-linked mortality [10].

As concern about the PRM grows, methods for control have become increasingly complicated. Traditional methods have relied on a range of acaricides, including carbamates, organophosphates, amidines, pyrethroids, and more recently spinosad, applied to premises and/or birds as sprays, mists and dusts [11–13]. Limitations of these approaches include: difficulties of achieving miticidal levels in all the hard-to-reach sites that harbour mites; additional stress to the birds from pesticide applications; risks of residues and pesticide exposure of workers; and the emergence of resistance to the available acaricides [11–17]. Therefore there is a long-recognized need for novel methods of reducing or even eliminating the threats that arise from PRM infestations [14].

Fluralaner, an isoxazoline compound for use as a systemic treatment for *D. gallinae* infestations, paralyses and kills mites through binding at a distinct, previously unrecognized receptor site on γ-aminobutyric acid (GABA)-gated and L-glutamate-gated chloride channels, which are widely expressed in insect and acarine central nervous and peripheral neuromuscular systems [18–20]. This mode of action is different from that of all other acaricides. A dose-ranging study in *D. gallinae-*infested birds demonstrated that two oral fluralaner administrations, 1 week apart, at a dose rate of approximately 0.5 mg/kg provided sustained mean mite count reductions of greater than 99% for 15 days following the first treatment, while a safety study in which birds were repeatedly treated at up to five times this dose demonstrated a wide safety margin [21, 22]. These preliminary experimental data and establishment of maximum residue levels to the satisfaction of regulatory authorities provided a basis for assessing the performance of fluralaner when administered under field conditions [23].

Studies were performed at 12 separate commercial production farms across France, Germany and Spain. The overall objective was to assess the safety and efficacy of a new fluralaner solution (10 mg/ml; 0.5 mg/kg body weight twice, 7 days apart) in the treatment of naturally acquired PRM infestations in chickens under a wide range of field conditions and management practices.

## Methods

The protocol was prepared in alignment with European guidelines for testing of antiparasitic products [24]. The studies were conducted in compliance with the Good Clinical Practice Guidance Document #85, May 9, 2001 (VICH GL9), and applicable regulatory requirements [25].

At each farm fluralaner efficacy was determined by the reductions in mean mite counts from traps placed in houses containing only fluralaner-treated birds in comparison with counts from traps placed in houses holding untreated control birds. Primary efficacy calculations were derived from two study phases: an initial phase to determine the onset of efficacy (up to 10 days duration) and a second phase in which assessments were concluded when a control group was treated for animal welfare or economic reasons.

Units for housing the birds were either separate buildings or rooms within a single building (Tables 1 and 2). Birds had access to free range on two farms. In each study all personnel, except the treatment dispenser and the farm manager who took no part in study assessments, were masked to treatment groups. At 11 of the 12 farms the studies were initiated during the months of the year (spring through summer) when ambient temperature and humidity favoured mite proliferation. On one farm the study began in October.

**Table 1:** Details of layer farms at the time of the first treatment

Farm	Housing	Breed	Birds/Unit	Age (weeks)
01-A	Barn, 2 rooms connected by a not-fully hermetic ceiling	Dekalb white	19,500	40
02-A	Barn with free range, 2 rooms, separated by a wall, connected by a not-fully hermetic door with 2 outdoor areas separated by a fence	ISA Brown	550	63 (C), 54 (T)
06-A	2 rooms, separated by a corridor. Enriched cages	ISA Brown	66,000	40
07-A	1 building, 2 rooms, separated by a corridor. Enriched cages	Novo Brown	62,000	36
09-A	2 separate buildings. Enriched cages	Lohmann Brown	100,000	22 (T), 27 (C)
DC1	2 separate buildings and free-range area	Lohmann brown	2714 (T), 2700 (C)	46
DC2	2 separate buildings. Enriched cages	Hy-Line brown	32,462 (T), 18,851 (C)	58
DC3	2 separate buildings. Enriched cages	Lohmann LSL	54,947 (T), 54,987 (C)	53 (T), 54 (C)

*Abbreviations*: *T* treatment group, *C* control group

**Table 2:** Details of replacement and breeder farms at the time of the first treatment

Farm	Housing	Breed	Birds/Unit	Age (weeks)
Replacement farms				
02-B	Barn, 2 rooms, separated by a corridor, 2 doors	Tetra Brown	4600 (T); 7500 (C)	13
02-C	Barn, 2 rooms, separated by a corridor, 2 doors	Tetra Brown, Harco Black, Koenigsberger Blue and Sussex	3000	11
Breeder farms				
04-A	Barn, 2 separate houses	Ross 308 (cocks and hens)	28,000	35 (T); 34 (C)
05-A	Barn, 2 separate houses	RJ344 (cocks) and Ross PM3 J (hens)	8200	41

*Abbreviations*: *T* treatment group, *C* control group

### Inclusion/exclusion criteria

For a farm to be included, units for each treatment group were required to be similar, to contain the same type of hybrid bird and to be comparable regarding flock size (exceptions were made at farms 02-B and DC2), age of birds, and management practices. Chickens had to be healthy and not suffering from any concurrent disease requiring treatment and to remain at the farm for a minimum of 3 months after Day 0, except for farms producing pullets (replacements) at which observations concluded after 42 days.

On all farms each study unit was required to have a proven infestation of *D. gallinae* mobile stages determined by mite counts from traps placed between Days -14 and -7 and collected 24 h after placement. Depending on the the housing arrangement and bird stocking density at each farm, the number of mobile *D. gallinae* stages (larvae, nymphs and adults) required to establish the presence of infestation in each unit ranged from 100 to 250 mites per trap in at least 50% of traps. This criterion was the same for each unit on any one farm.

Flocks could not have been treated with products effective against *D. gallinae* in the 8 weeks before Day 0, or in the last month with non-pharmaceutical products. Acaricide treatments with potential efficacy against *D. gallinae* were not permitted during the study unless indicated for animal welfare reasons.

### Study procedures

At each farm, personnel involved in any study activities were instructed to change all handling equipment and clothing for any movement between units, and to wear single-use overall and overshoes or similar whenever entering a study unit. All working equipment was to be available in double, one set for each unit.

Feed and drinking water provision, air conditioning and stocking density followed the routine procedure of the farm. Study chickens were provided periodic veterinary care according to farm procedure, and general health observations were conducted daily in each study unit from Day -1 to Day 2, on Days 6 to 9, and then weekly until the end of the study. On study days without veterinary health observations, the farm manager documented the general impression of the flock and was to advise the dispenser immediately upon observation of any health abnormalities in study chickens.

### Treatment

With the exception of farm 02-B, the unit with the highest mite infestation was allocated to the fluralaner group, the other unit to a control group that was to remain untreated for mites (unless rescue treatment was required for animal welfare or economic reasons). The targeted fluralaner dose rate was 0.5 mg/kg administered twice, with a seven-day interval between administrations (Days 0 and 7), corresponding to 5 ml of fluralaner per 100 kg total body weight. Medicated water was freshly prepared and administered via a medication tank or using a dosing pump.

At each farm, within the week preceding Day 0, the inner surfaces of the drinking water distribution system of the fluralaner-treated unit were cleaned. Drinking water consumption in each of the units allocated to the fluralaner group was determined on 1 day between Days -3 and -1 (and also on Day 6 at replacement farms).

To estimate the total flock body weight to be treated, on Days -1 or -2, 24 chickens were randomly selected from each unit to be medicated and weighed. Whenever cocks were present, the proportion of hens and cocks selected for weighing was representative of the broader population in each unit. The average chicken body weight and total flock body weight were then calculated based on records of the number of chickens in the flock on each day of fluralaner administration.

Immediately prior to fluralaner administration, except at farm DC2 because of risk of leakage, drinker lines were emptied, and at some farms flushed. Medicated water was prepared on each treatment day using a specific volume of the fluralaner solution (10 mg/ml) calculated according to the total estimated body weight of chickens to be treated multiplied by 0.05 (to achieve the targeted dose of 0.5 mg/kg). The product was mixed with water in a medication tank or in a stock-solution container when a dosing pump was available. Medicated water was then dispensed continuously until the medication source was empty. Visual inspection of the medicated water supply and drinking nipples verified that there were no medication obstructions or irregularities at any farm. Once empty, the medication sources were rinsed with unmedicated tap water. The pipes were then connected to the regular water distribution system to completely displace the remaining medicated water in the system. The tap-water rinse was then provided to the fluralaner-group birds.

### Production parameters

 The number of collected eggs was recorded daily for laying and breeding hens. Records were also available for the percentage of downgraded eggs at farms DC2 and 09-A and egg hatchability at the two breeder farms (04-A, 05-A).

All study chickens that died or were culled between Day 0 and the end of the animal phase at a farm were collected and, if possible, stored in a deep freezer. If the weekly mortality rate exceeded the usual weekly mortality rate of each unit, all cadavers or examiner-selected cadavers, were necropsied by a qualified veterinarian.

### Mite counts

**Fig. 1:**
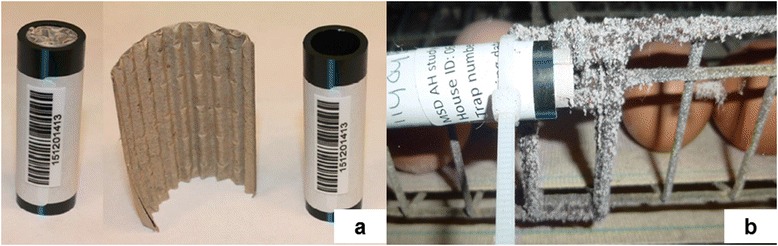
Mite traps. **a** Black polyethylene tubes with inner and outer diameters of 12 mm and 16 mm, respectively, containing rolled corrugated 50 × 60 mm cardboard with thickness of 1 mm (Avivet, the Netherlands). **b** Example of attachment of a mite trap in an area of mite aggregation

The mite infestation level in each unit was determined using eight to 24 traps (Fig. 1a) depending on flock and house size and production type, placed between Day -14 and -7, depending on farm, and on Days -1, 0 or 1, 3, 6, 9, and 13 or 14, then at weekly or two-weekly intervals until the end of the assessment phase. Traps were evenly distributed throughout units, with a similar distribution of traps in each corresponding unit. Traps were placed close to potential areas of mite aggregates, at fixed positions throughout the study, out of reach of the chickens, and fastened horizontally as far as possible from air ventilation systems (Fig. 1b). Traps were collected 24 h after placement and deep frozen for at least 48 h prior to shipment, or placed on dry ice for immediate shipping to a central laboratory where they were opened for counting and stage differentiation.

The mites in each trap and its plastic bag were poured into a Petri dish. Remaining mites or eggs on the cardboard of the trap or in the plastic bag were added to the mites in the dish. Mite stages were identified, differentiated and counted [26]. In traps with up to 250 mg of *D. gallinae* (eggs and mobile stages), all mites were differentiated and counted. For traps containing more than 250 mg of mites, a subsample of approximately 100 mg was used for differentiation and counting.

### Adverse events

An adverse event (AE) was any observation, whether or not considered product-related, that was unfavourable and unintended and occurred after the use of fluralaner. Other conditions (including mortality) commonly associated with commercial poultry husbandry (e.g. feather pecking, low level lameness, wounds, etc.) were expected and not considered to be AEs.

### Efficacy assessment

The statistical units for antiparasitic efficacy evaluation were mite traps, and for production parameters the experimental unit was the poultry unit. Homogeneity of study groups was evaluated descriptively for the distribution of Day -1 *D. gallinae* counts in each study group and production type. The statistical analysis was performed by means of the software package SAS^®^ (SAS Institute Inc., Cary, NC, USA, release 9.2).

The determination of primary efficacy was based upon the *D. gallinae* counts in traps collected from the units with fluralaner-treated birds compared with the control units. Percent efficacy was calculated separately for each farm for each post-treatment assessment time point using the Henderson-Tilton formula:

$$ \mathrm{Efficacy}\ \left[\%\right]=\left(1\hbox{-} \frac{{\mathrm{T}}_{\mathrm{post}}}{{\mathrm{C}}_{\mathrm{post}}}\times \frac{{\mathrm{C}}_{\mathrm{pre}}}{{\mathrm{T}}_{\mathrm{pre}}}\right)\times 100 $$

where T_post_ is the mean number of mites per trap in a unit with treated chickens for each post-treatment time point; C_post_ is the mean number of mites per trap in the control unit for each post-treatment time point; T_pre_ is the mean number of mites per trap in a unit with treated chickens on Day -1; C_pre_ is the mean number of mites per trap in the control unit on Day -1. The mean number of mites denotes the arithmetic mean of all mobile stages, i.e. larvae, nymphs (both stages together) and adults. Fluralaner was considered effective at a certain time point if efficacy was at least 90%.

Chicken mortalities (including culls) in each unit during the study were compared to the pre-treatment mortalities of each group and summarized. The weekly laying rate was calculated as the number of eggs collected in relation to the number of chickens in the unit. Pre-treatment production parameters (weekly means) of each unit were compared to the post-treatment parameters, and the percent changes in treated chickens were compared to those in control chickens.

## Results

There were eight layer farms, two breeding farms and two replacement farms enrolled into the studies. The breeds and ages of birds in each group (treated or control) were the same or generally similar within each farm, with the greatest difference in age of birds at a layer farm (02-A) (Tables 1 and 2).

### Mite count reduction

**Fig. 2:**
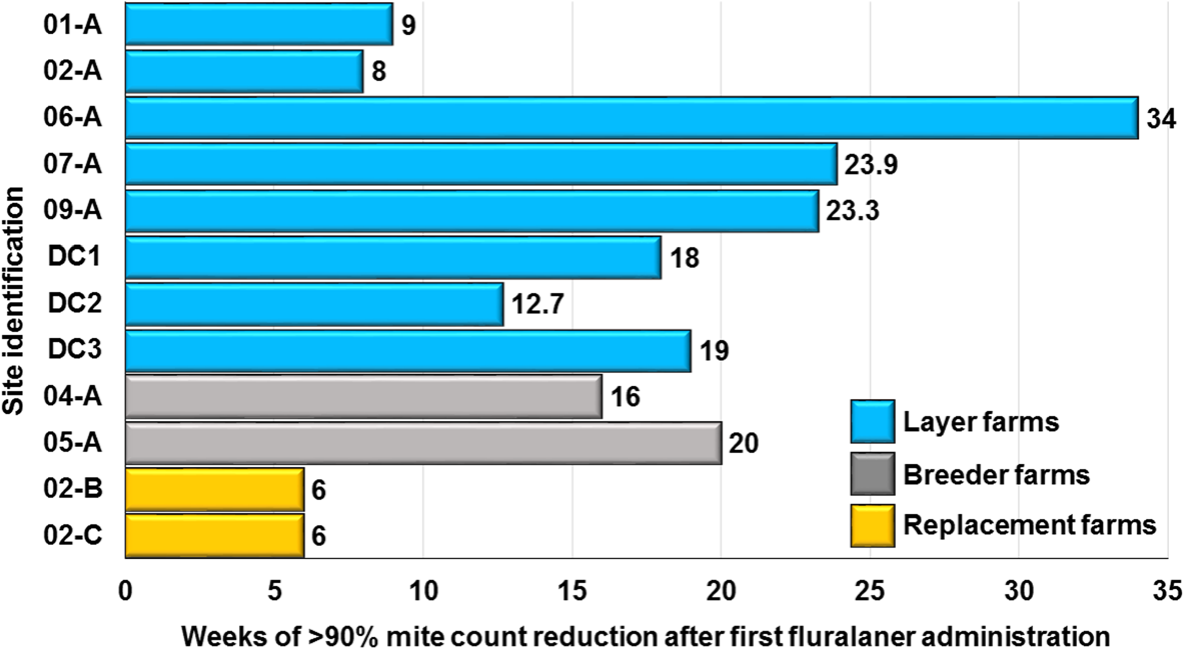
Duration of mite population control in fluralaner-treated units per farm (last timepoint with mite reduction > 90%). Sites 01-A and 02-A had inadequate separation of the treatment groups, resulting in increased risk of mite cross-contamination between units, leading to termination of study assessments. At farms 06-A, DC2, 05-A, 02-B and 02-C assessments were concluded at the end of the production cycle or transfer of chickens to another farm

**Fig. 3:**
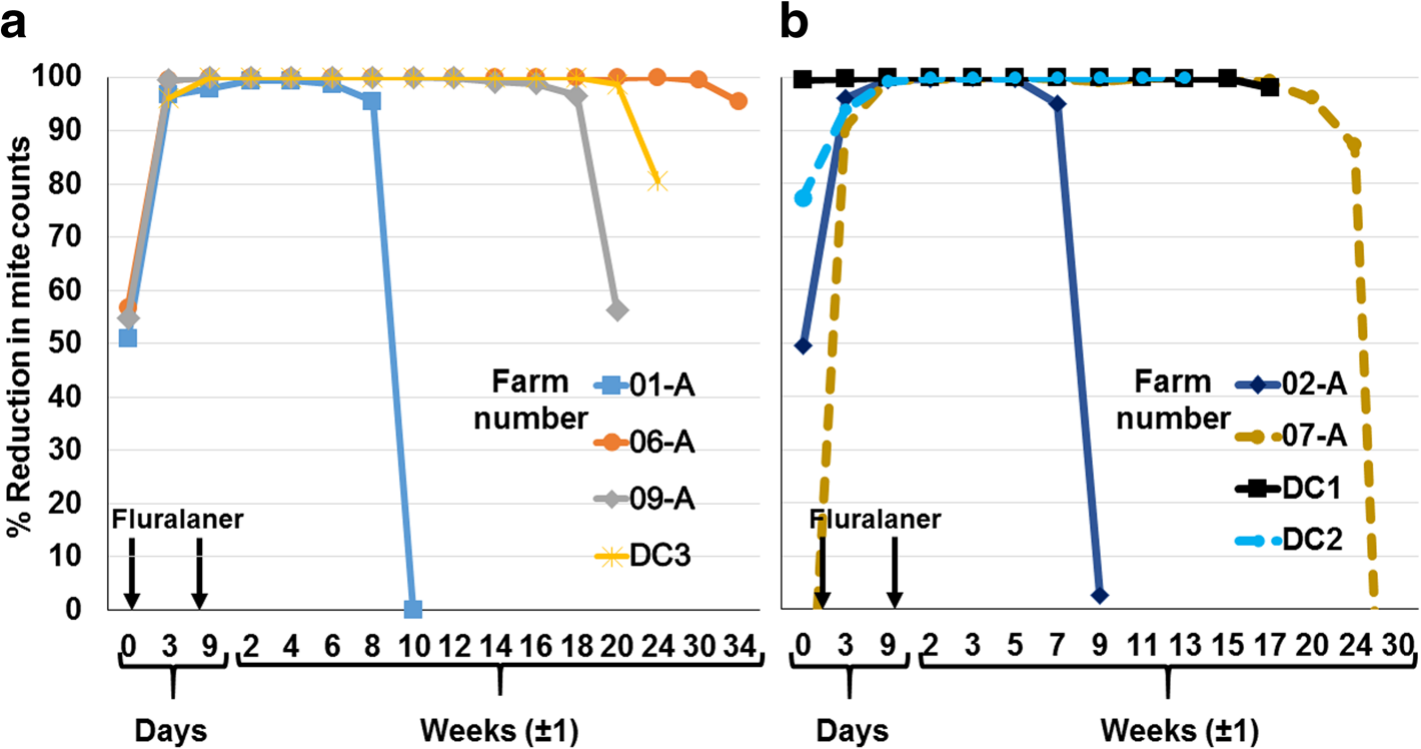
Mite count reductions from fluralaner-treated units at layer farms. **a** Sites at which rescue-treatments were administered to control units. **b** Sites that did not have rescue-treatment of control birds

On all sites, by Day 14 efficacy was greater than 99%, remaining at a level of at least 90% through the end of the production cycle (approximately three to 8 months, farms DC2 and 06-A); until mite regrowth in the treated unit (approximately two to 6 months, farms DC3, 01-A, 02-A, 07-A, 09-A), or until the study was terminated because of declining mite counts in the control unit (4 months, DC1) (Tables 3 and 4; Fig. 2). For animal welfare reasons, a supplementary acaricide treatment (spinosad; Elector^®^, Elanco) of the control unit was provided on Week 4 on Farm 06-A, Week 9 on Farm 01-A, and Week 20 on Farm 09-A, and (a non-chemical product) on Farm DC3 on Weeks 11, 12 and 15. Despite these acaricide treatments, mite counts from control units remained at levels that were sufficiently high to allow extended comparisons with the fluralaner-treated group beyond the dates of these treatments. These extensions were then concluded at either the end of the production cycle (DC3, 06-A) or when mite regrowth was apparent in traps from the treated unit (01-A, 09-A). On this basis, mean mite count reductions in the units in which birds received fluralaner continued to exceed 90% (up to 100%) for at least 56 days and up to 238 days (Tables 3 and 4; Fig. 3a). The post-Day 56 decline in effectiveness at Farms 01-A and 02-A was attributed to inadequate separation of the fluralaner-treated group units from the respective control units, leading to a rapid re-infestation of the treated unit (Fig. 3a, b).

**Table 3:** Initial mite counts per unit and percentage mite efficacy on layer farms

Farm	Mean mite counts, Day -1		Efficacy (%) on days after the first fluralaner administration					
	Treated	Control	0	1	3	9	14	Last day with > 90% efficacy
01-A	568	409	50.7	nd	96.6	97.8	99.3	90.7 (Day 63)
02-A	2138	1673	74.8	nd	98	99.7	99.8	97.5 (Day 56)
06-A	2245	758	56.8	nd	99.4	100	100	95.4 (Day 238^a^)
07-A	196	162	26	nd	95.3	100	99.8	93.6 (Day 167)
09-A	979	694	54.9	nd	99.4	100	100	96.5 (Day 126)
DC1	2250	751	–	99.7	99.8	100	100	100 (Day 119)
DC2	1610	367	–	88.7	96.9	99.6	99.9	100 (Day 89^a^)
DC3	1313	1194	–	nd	96.0	99.9	99.9	98.7 (Day 133)

*Abbreviation*: *nd* not determined

^a^End of the production cycle

**Table 4:** Initial mite counts per unit and percentage mite efficacy on replacement and breeder farms

Farm	Mean mite counts, Day -1		Efficacy (%) on days after the first fluralaner administration				
	Treated	Control	0	3	9	14	Last day with > 90% efficacy
Replacements							
02-B	430	998	69.2	95.3	100	99.7	96.0 (Day 42^a^)
02-C	1974	347	15.1	96	99.9	100	99.6 (Day 42^a^)
Breeders							
04-A	1148	521	71	98.9	100	100	94.5 (Day 112)
05-A	9009	3364	65.2	99.8	100	100	99.9 (Day 140^a^)

^a^End of the production cycle or transfer to another farm

**Fig. 4:**
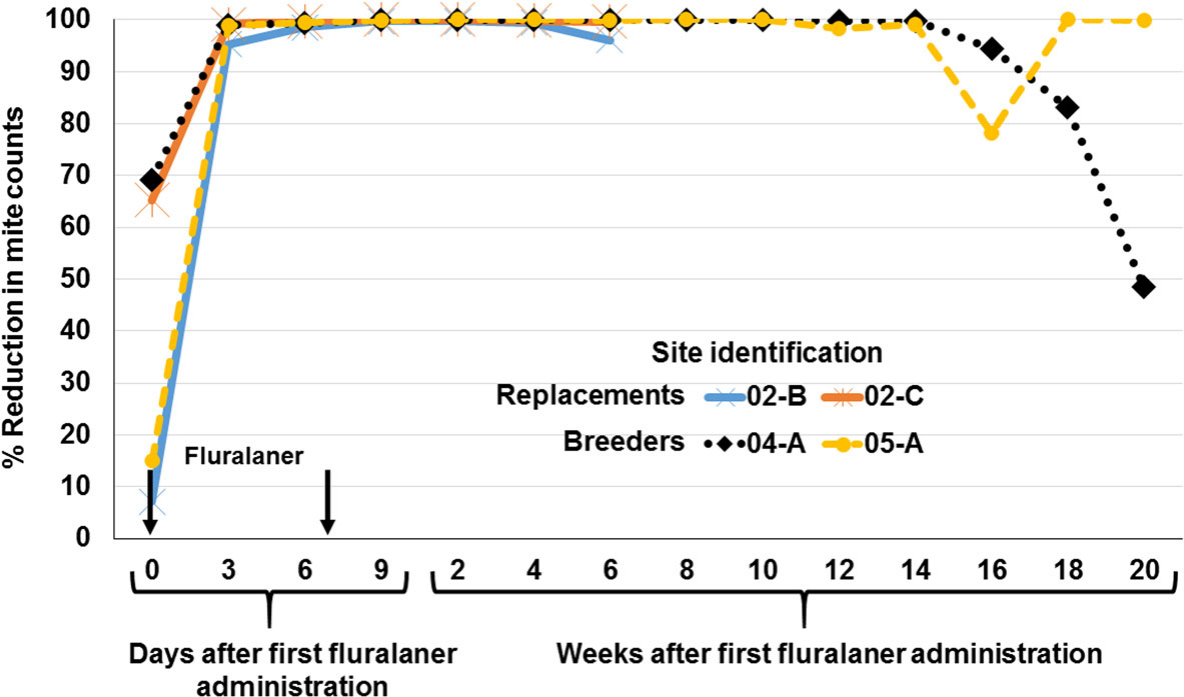
Mite count reductions from fluralaner-treated units at the replacement (pullet) and breeder farms. The decline in efficacy at Week 16 on site 5-A was attributed to a decline in mite counts in the control group, rather than being caused by a resurgence in mite population in the unit housing fluralaner-treated chickens

At the two breeder farms, in the units in which birds were treated with fluralaner the mite reduction exceeded 99% from Day 6 until Week 14. At Farm 04-A this reduction in mite counts was 100% on Weeks 5 through 8, and on Farm 05-A was 100% at Weeks 2, 4, 8, 10 and 18 (Fig. 4). Mean mite trap counts increased in the treated unit at Farm 04-A after Week 16, while high efficacy was maintained until the end of the study in Week 20 (Day 140) at farm 05-A. At the two replacement chicken (pullet) farms the fluralaner treatment resulted in the mite reduction exceeding 95% from Day 3 until end of the study at week 6 (Day 42) (Fig. 4).

Hatched eggs of *D. gallinae* produce motile but non-feeding larval stages which were also assessed from the collected traps. Across farms, overall reductions in larvae detected in traps had been reduced by 76.9% by Day 3 and 99.9% by Day 9.

### Production assessments

Across all study farms, the weekly mortality rates were comparable between treatment groups before and after Day 0. At one farm (DC1) there was a 0.04% increase in mortality in the fluralaner-treated group due to a colibacillosis outbreak, diagnosed at necropsy and by bacteriology.

In layers allocated to the fluralaner group, pre-treatment mean weekly laying rate ranged from -5.8% lower to 4.1% higher than in hens allocated to the control group (Table 5). Post-treatment improvement in egg laying was greater in the fluralaner-treated group at all layer farms, with the difference from the control group ranging from 0.9 to 12.6%. On the two breeder farms, the difference for fluralaner compared to controls was -2.2% and 5.7%. An observation reported from layer farms was that control-group chickens would sometimes avoid laying eggs in infested nests. At farms DC2 and 09-A, the percent of downgraded eggs was also recorded, with an overall post-treatment compared to pre-treatment improvement in the treated group of 3.4% and 1.1%, respectively.

**Table 5:** Difference in average weekly laying rate (treated - control) pre- and post-treatment on layer and breeder farms

	Farm	Pre-treatment (%)	Post-treatment (%)	Difference (%)
Layers	01-A	+0.43	+1.33	+0.90
	02-A	+4.05	+16.63	+12.58
	06-A	+0.08	+1.15	+1.07
	07-A	-0.58	+1.45	+2.03
	09-A	-1.00	+4.70	+5.70
	DC1	-0.40	+0.50	+0.90
	DC2	-1.30	-0.40	+0.90
	DC3	+3.20	+5.10	+1.90
Breeders	04-A^a^	-1.08	-3.24	-2.16
	05-A	-5.83	-0.16	+5.67

^a^Although the units were comparable in protocol designated characteristics, the control unit had a history of better performance relative to the treated unit

At Farm 05-A, the overall hatchability rate over the complete laying period was higher in the fluralaner-group unit (78.7%) than in the control unit (75.8%). At the other breeder farm (04-A), the difference in hatchability rates between the treated and control groups was identical pre- and post-treatment.

Apart from a colibacillosis outbreak at farm DC1 7 weeks following fluralaner treatment initiation, there were no abnormal general health observations in treated birds. There were no treatment-related AEs.

## Discussion

Parasitic stages of *D. gallinae* spend only about 1 hour obtaining a blood meal and approximately 23 h in the environment, so that removing traps 24 h after placement would allow adequate time for trap counts to be a reliable indicator of the level of challenge in a poultry house. The trap methodology has been reported previously and has been validated as a robust method to quantify mite infestations of poultry, showing that the number of mites collected in traps can be statistically correlated to the mite population in a cage [27, 28].

Once collected, the traps were deep frozen to ensure that the mites were killed, thereby avoiding the formation of mite agglomerates that would have made counting unreliable. The resulting mite counts demonstrate that a substantial mite challenge of the untreated control birds was maintained throughout all but one of the study farms, despite, in a number of cases, repeated acaricide spray treatments.

The 90% efficacy threshold selected to determine the duration of mite population control aligns with efficacy thresholds accepted by veterinary regulatory agencies for assessing ectoparasite control products [24]. On this basis, the mite population control (i.e. > 90% reduction in mite counts) provided by the fluralaner treatment was of a rapid onset, almost complete and sustained for an extended period on each farm, regardless of production type or drinking water system. In every treated unit there was a dramatic reduction of up to 100% in mite counts.

The major factor contributing to the resurgence of mite counts in units with the chickens receiving fluralaner was the reliability, or lack thereof, of the separation of the treated and untreated groups. The presence of a non-hermetic door, fence, or ceiling was associated with a faster mite regrowth in the treated unit than when the groups were more substantially separated, as in units in two separate buildings. Because of the protocol requirement for an untreated control unit in close proximity to a treated unit, movement of study personnel and equipment, including conveyors, between the 2 units (regardless of best efforts at protocol adherence and the rigour with which hygiene was practiced) would have greatly increased the risk of mite cross-contamination to the treated units. The results reported here therefore represent a worst-case scenario, as under normal commercial conditions all houses or rooms which are adjoining or in close proximity would be treated simultaneously, thereby substantially reducing or eliminating this risk of cross-contamination.

The rapid removal of adults from the population prevents additional egg production by female mites. As eggs that were present before treatment hatch within two to 3 days under favorable conditions, the emerging larvae are progressively killed after they mature to the nymphal stages and begin feeding [14]. The second fluralaner administration, 1 week after the first, kills any mites originating from eggs present at the time of the first treatment. Therefore the fluralaner treatment regimen provides the opportunity to provide a substantial and sustained reduction, or total elimination of mites from a production system. Moreover, as the PRM prevalence in laying hens is at least 80% in most of the major European egg-producing countries [7], the treatment of pullets just before they are transferred may be a valuable tool in eliminating an important source of reinfestations, and in reducing the overall prevalence of PRM in layer facilities.

The results in our studies are consistent with the linkage of PRM infestation with welfare and productivity losses, as the acaricidal efficacy of fluralaner and the resulting relief of mite-infested birds was reflected by an overall increase in laying rate in the treated groups, and a reduction in the percentage of downgraded eggs. An interesting finding was that in some cases, under conditions of mite challenge layers avoided their nests. Additionally, increased hatchability rates were apparent in the treated group at one of the two breeder farms. These numerical improvements in laying rates appear sufficiently promising to generate further investigation into the production benefits of fluralaner medication of PRM-infested poultry farms.

This is the first study in which the potential value of an isoxazoline has been demonstrated in food-producing animals, and the first report of successful elimination of *D. gallinae* from birds maintained under commercial conditions. Administration of fluralaner to the birds in this study followed the establishment of maximum residue limits for fluralaner that were adopted by the European Commission [23].

Consistent with findings from the use of fluralaner in dogs and cats, the tolerability of the fluralaner treatment in the present set of studies was excellent. There were no treatment-related AEs following the treatment of approximately 355,000 chickens across 12 farms.

## Conclusion

The administration of fluralaner solution (10 mg/ml) given orally via drinking water at a dosage of 0.5 mg/kg body weight on two occasions with a seven-day interval, was well tolerated and highly effective against the poultry red mite (*D. gallinae*) in naturally infested chickens across a range of production types and management systems. By Day 14, mite count reductions in fluralaner-treated birds was greater than 99%, continuing at a reduction of least 90% for up to 8 months after treatment. The results indicate that this formulation of fluralaner has potential to be the cornerstone of an integrated approach to reducing or eliminating the welfare and productivity costs of this increasingly threatening pest.

## Abbreviations

AE: adverse event
C: untreated control group
C_post_: mean number of mites per trap in a control unit for each post-treatment time point
PRM: poultry red mite (*Dermanyssus gallinae*)
T: fluralaner-treated group
T_post_: mean number of mites per trap in a unit with treated chickens
VICH: International Cooperation on Harmonization of Technical Requirements for registration of Veterinary Medicinal Products
